# Links between fear of humans, stress and survival support a non-random distribution of birds among urban and rural habitats

**DOI:** 10.1038/srep13723

**Published:** 2015-09-08

**Authors:** Natalia Rebolo-Ifrán, Martina Carrete, Ana Sanz-Aguilar, Sol Rodríguez-Martínez, Sonia Cabezas, Tracy A. Marchant, Gary R. Bortolotti, José L. Tella

**Affiliations:** 1 Departamento de Ecología, Genética y Evolución & IEGEBA-CONICET, Facultad de Ciencias Exactas y Naturales, Universidad de Buenos Aires, Buenos Aires, Argentina; 2 Department of Physical, Chemical and Natural Systems, Universidad Pablo de Olavide, Sevilla, Spain; 3 Department of Conservation Biology, Estación Biológica de Doñana, CSIC Sevilla, Spain; 4 Instituto Mediterráneo de Estudios Avanzados, IMEDEA (CSIC-UIB), Miquel Marqués 21, E-07190 Esporles, Islas Baleares, Spain; 5 Department of Biology, Biochemistry and Pharmacy, Universidad Nacional del Sur, Bahía Blanca, Argentina; 6 Department of Biology, University of Saskatchewan, 112 Science Place, Saskatoon, SK S7N 5E2, Canada

## Abstract

Urban endocrine ecology aims to understand how organisms cope with new sources of stress and maintain allostatic load to thrive in an increasingly urbanized world. Recent research efforts have yielded controversial results based on short-term measures of stress, without exploring its fitness effects. We measured feather corticosterone (CORT_f_, reflecting the duration and amplitude of glucocorticoid secretion over several weeks) and subsequent annual survival in urban and rural burrowing owls. This species shows high individual consistency in fear of humans (i.e., flight initiation distance, FID), allowing us to hypothesize that individuals distribute among habitats according to their tolerance to human disturbance. FIDs were shorter in urban than in rural birds, but CORT_f_ levels did not differ, nor were correlated to FIDs. Survival was twice as high in urban as in rural birds and links with CORT_f_ varied between habitats: while a quadratic relationship supports stabilizing selection in urban birds, high predation rates may have masked CORT_f_-survival relationship in rural ones. These results evidence that urban life does not constitute an additional source of stress for urban individuals, as shown by their near identical CORT_f_ values compared with rural conspecifics supporting the non-random distribution of individuals among habitats according to their behavioural phenotypes.

Urbanization is one of the most prevailing and lasting forms of habitat change occurring worldwide and causing the loss of biodiversity through local extinction processes^1^. Nevertheless, species greatly vary in their responses to these severe changes in the environment; while most are unable to occupy these new habitats (termed as urban avoiders), others are able to persist (urban adapters) or even reach higher densities in urban than in rural areas (urban exploiters)^2^,^3^. This interspecific variability has encouraged the study of the ecological and life history traits that allow some species to thrive in urban environments. Using birds as study subjects, recent comparative works have successfully related variability in environmental tolerance^4^, life-history traits^5^ and inter-individual variability in behavior^6^ to the different responses of species to urbanization. In addition to interspecific approaches, research on how individuals respond to and cope with urbanization as well as on the associated physiological and behavioral mechanisms should help us to understand the observed patterns^7^,^8^. Challenges and opportunities associated with urbanization include changes in resource availability^9^, predation risk^10^, conspecific density^11^, new competitors^12^ and human disturbance^6^, as well as other perturbations such as light pollution and the artificial elevation of natural noise levels (e.g.^13^,^14^). All of these factors are potential causes of stress that may affect the fitness of individuals, and how they are able to deal with stress may explain their ability to cope with urban challenges and opportunities and, ultimately, the different responses of species to urbanization^7^.

To properly understand whether urbanization represents an opportunity or a challenge for individuals, it is essential to understand how individuals actually perceive their environment. The endocrine system is a primary candidate for this purpose, given its role in mediating the physiological and behavioral responses of organisms to the environment. Recent efforts, within the nascent field of urban endocrine ecology, have been devoted to understanding the functional role of the hypothalamic–pituitary–adrenal (HPA) axis in mediating responses to urbanization^7^. Measures of glucocorticoids - mostly baseline corticosterone (CORT) and stress-induced CORT - have been used to estimate the allostatic load (i.e., the cumulative current and anticipated energetic demands on an organism) experienced by urban and rural individuals of the same species^7^. More than 20 studies, mostly on birds, have been conducted in recent years; however, no consistent patterns have been revealed for differences in allostatic load between urban and rural conspecifics^7^. The fact that similar proportions of studies showed higher, lower, and similar circulating CORT levels in urban vs. non-urban birds may be related to methodological issues (the short-term nature of blood CORT measurements and variability in the life stages of individuals measured), but also to the fact that challenges faced by some species in urban habitats may represent opportunities for others^7^. Therefore, more research is needed to understand the changes in allostatic load of individuals associated with urban life, as well as their uninvestigated fitness consequences of these changes^7^.

Here, we deal with two open questions highlighted by Bonier^7^ in her recent review: 1) Do hormonal responses regulate behavioral adaptation to urban environments, such as reduced flight initiation distances (FID)? and 2) Are hormone-fitness relationships affected by urbanization? We approach these questions in the light that modulated sensitivity of the HPA axis may play a central role in allowing urban birds to maintain foraging, breeding, and other essential behaviors in the face of frequent exposure to human disturbance^7^. Studying a contemporary process of urbanization, Carrete & Tella^6^ found that inter-individual variability in the fear of humans (experimentally measured as FID of individuals when approached by a human) could explain why some species thrive successfully in urban habitats. We focus here on one of these species, the burrowing owl (*Athene cunicularia*), an urban exploiter which shows higher abundance and density in urban than in nearby rural habitats^6^,^11^. Burrowing owls showed high within-individual consistencies in FID throughout their lifespan in both urban and rural habitats (repeatability > 0.90^15^), suggesting that the lower FID of urban birds does not arise from habituation but from the selection of individuals able to cope with constant human disturbance^6^,^16^. Thus, if individual’s behavioural phenotypes match well with the differential levels of human disturbance in the areas where they settle to breed -as posit by the human disturbance habitat selection^17^ and the habitat-matching dispersal^18^ hypotheses, urban birds should not perceive their habitat as more stressful than that of rural ones. We therefore predict no differences in the allostatic load between urban and rural individuals nor a relationship with their FIDs. The majority of studies utilizing levels of CORT as indicator of the allostatic load of individuals have been conducted using instantaneous blood samples, which can be particularly informative when characterizing the functioning of the HPA response and understanding an individual’s physiological state at the instant of sampling. However, the time frame of such samples is limited, so integrated measures of glucocorticoids have been developed and applied to ecological contexts^19^, notably CORT in feathers (CORT_f_). This biomarker, whose time frame is significantly longer than that of blood, has been related to diverse ecological factors, suggesting that it integrates CORT secretion in general, globally expressing the response of individuals to different sources of environmental variation^20^. Importantly, CORT levels increase in feathers when individuals are exposed to long-term stressors^21^, as could be expected if urban life actually represents a sustained source of stress for urban individuals. It has been also suggested that CORT_f_ could be used as a metric to study carry-over effects^22^. In fact, two recent studies showed links between CORT_f_ and survival in two bird species^23^,^24^. Therefore, we examined the relationships between CORT_f_ levels and subsequent survival, predicting a negative relationship^23^ according to the corticosterone-fitness hypothesis^25^ independent of the habitat in the area where they settled. For these purposes, we individually marked a number of urban and rural breeding owls, measured their FID and CORT_f_, and closely monitored them for their survival until the next breeding season. Our results fully support our first predictions, but also show complex relationships between CORT_f_ and survival that may be related to ecological constraints (i.e, predation pressure) other than human disturbance.

## Results

### Fear of humans and stress

**Figure 1:**
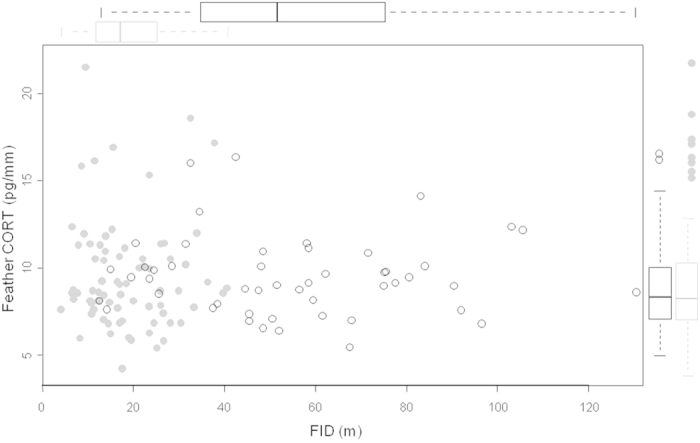
Relationships between feather CORT (CORT_f_) and FID of urban (grey dots) and rural (white dots) adult burrowing owls. In the external margins of the plot, we included the boxplots for FID and CORT_f_ of rural (n = 49) and urban (n = 72) birds.

According to previous studies, urban burrowing owls showed significantly shorter FIDs than their rural conspecifics (urban birds: mean = 18.29 m, SE = 1.05, n = 72; rural birds: mean = 54.51 m, SE = 3.83, n = 49), even while controlling for the slightly shorter -although not statistically significant- FIDs in males (mean = 31.19 m, SE = 3.49, n = 56) than in females (mean = 34.48 m, SE = 3.12, n = 65) (Table 1). However, this closer interaction with humans does not suppose an extra-source of stress, as urban birds showed nearly identical CORT_f_ concentrations than rural ones (urban birds: mean = 9.61 pg/mm, SE = 0.38, n = 72; rural birds: mean = 9.57 pg/mm, SE = 0.33, n = 49, Fig. 1). CORT_f_ did not differ between habitat types even when controlling for year and the slightly and marginally significant lower CORT_f_ concentrations found in males (mean = 9.01 pg/mm, SE = 0.29, n = 56) than in females (mean = 10.10 pg/mm, SE = 0.40, n = 65; Table 1). Moreover, CORT_f_ concentrations were not related to individual FIDs, either when considering all birds together (Table 1) or when taking into account potential differences between habitats (interaction habitat *FID: F_1,115_ = 0.05, p = 0.82; Fig. 1).

### Stress and survival

**Figure 2:**
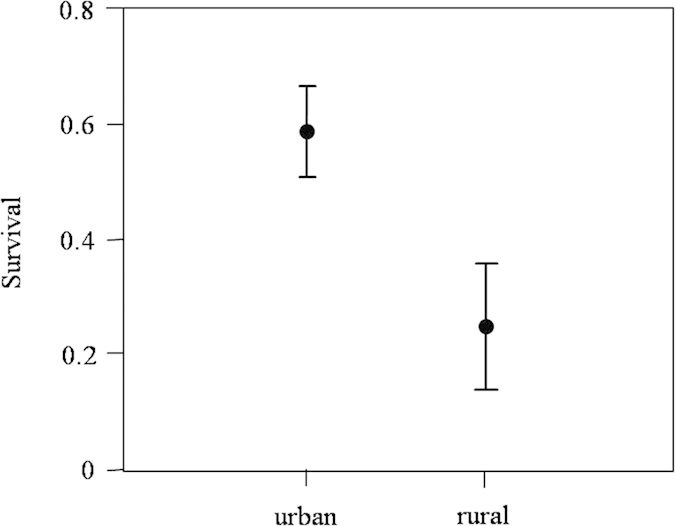
Estimates of annual survival (mean and 95% CI) obtained for 109 urban and 74 rural adult burrowing owls.

The goodness-of-fit of the general model fitted the data adequately (χ^2^ = 2, df = 5, p = 0.85). We started model selection by testing the effects of time and habitat type on resighting (*p*) and survival (ϕ) probabilities (Table 2). The best model (Model 1, Table 2) included a temporal effect on resighting probability, which was relatively high (*p*_mean_ = 0.81, 95% CI = 0.68–0.89) and did not vary between habitats. However, survival differed between habitats, being more than twice as high in urban (ϕ = 0.59) as in rural (ϕ = 0.25) individuals (Fig. 2). Models including additional temporal effects on survival had ΔAICc > 2 and much lower Akaike weights (Table 2). Consequently, we selected the structure of this model to test for CORT_f_ effects on subsequent annual survival.

**Figure 3:**
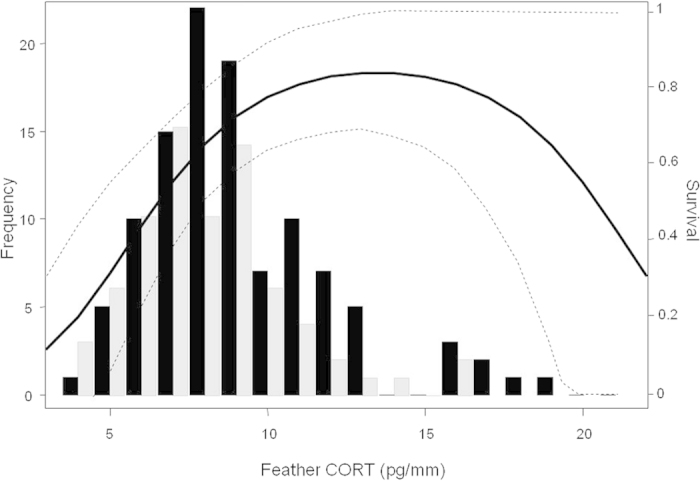
Distribution of feather CORT (CORT_f_) concentrations measured in urban (n = 109, black bars) and rural (n = 74, grey bars) burrowing owls. Overlapped, we show the relationship between CORT_f_ and the estimated immediate survival of urban owls (black line, 95% CI depicted as dashed lines). There was not a significant relationship for rural individuals (see Results).

The effect of CORT_f_ on survival was retained in model selection (Table 3). The best supported model in terms of AICc (Model 1a, Table 3) showed that survival decreased with CORT_f_ levels in rural birds (logit ϕ_rural_ = 1.851 (CI: −1.846–5-548)–0.382 (CI: −0.815–0.052)*CORT_f_) while it exhibited a quadratic relationship in urban ones (logit ϕ_urban_ = −4.547 (CI: −8.424–−0.671) + 0.917 (CI: 0.189–1.644)*CORT_f_–0.034 (CI: −0.065–−0.004)*CORT_f_^2^). However, the confidence interval for the beta estimate corresponding to the linear slope of rural birds included zero, indicating a non-significant effect. In fact, a more parsimonious model without this effect was equivalent in terms of AICc (ΔAICc = 1.90, Table 3), indicating that CORT_f_ had a non-detectable effect on the survival of rural birds but a significant quadratic relationship in urban owls (logit ϕ_urban_ = −4.646 (CI: −8.493–−0.798) + 0.927 (CI: 0.205–1.650)*CORT_f_–0.034 (CI: −0.065–−0.004)*CORT_f_^2^). Accordingly, ∆AICc between models 1b and 1i (no CORT_f_ effect on survival of rural and urban birds) was 5.38 (Table 3), supporting the CORT_f_-survival relationship in urban birds (Fig. 3). Therefore, in addition to habitat differences, CORT_f_ had a significant effect on the survival of urban birds, with probabilities of survival being higher at intermediate CORT_f_ levels (Fig. 3).

## Discussion

### Human disturbance and stress in urban and rural birds

With the accelerating pace of anthropogenic global change, understanding how organisms cope with these changes is an urgent task. Traditionally, the most adverse human activities for fauna have been related to natural landscape modification. However, a growing number of studies are now showing that human presence *per se* can be important in modifying animal activities and distributions through behavioral changes^7^,^21^. Indeed, it has been proposed that the ability of certain species to thrive in urban habitats, the most populated habitats worldwide, can be attributed to their high interindividual variability in fear of humans, such that only tame individuals (i.e., those with the lowest fear of humans compared to their conspecifics) can successfully occupy these areas^6^,^15^,^17^. Our result showing shorter FIDs in urban than in rural burrowing owls are thus not novel, but in line with previous studies on this species^15^,^17^ and with others showing the same pattern of higher tolerance towards humans in urban individuals in a variety of birds species^16^. Although this pattern was considered a result of habituation to human disturbance in the absence of individual-based studies^26^, the long-term monitoring of individually marked burrowing owls demonstrated that FID remains largely unchanged across their lifespan^15^, thus supporting the idea that behavioral differences between urban and rural conspecifics are more likely a consequence of selective processes^6^,^16^,^27^. Urbanized areas may be selecting for individuals with coping styles able to deal with urban challenges, human presence included^27^,^28^,^29^,^30^,^31^. Coping styles (so called behavioral syndromes or personalities) are relatively stable combinations of behavioral and physiological traits that confer differential fitness consequences under divergent environmental conditions^32^. Different studies have demonstrated a genetic basis for the expression of behavioral and physiological components of individual coping styles^33^, such that selective pressures associated with urban life can have long-lasting ecological but also evolutionary consequences. In fact, a recent study on urban and rural dark-eyed juncos (*Junco hyemalis)* supports the role of selection instead of drift or founder effects to explain the lower FID shown by urban birds^27^.

We aimed here to determine whether hormonal responses of individuals to stress are related to their tolerance to human disturbance, to deepen our understanding of the potential selection of individuals able to cope with urban habitats. Attending to the above arguments, we should not expect a relationship between FID and CORT_f_ nor differences in CORT_f_ between urban and rural individuals. If individuals choose to breed in urban habitats^6^ or in rural sites with different levels of human disturbance according to their tolerance to humans^17^, all individuals should perceive similar amounts of stress in regards to human disturbance. Our results fully support these predictions. However, they may also be interpreted as another piece to the puzzling results obtained to date, which cannot discern a clear pattern of differences in allostatic load between urban and rural birds^7^,^34^. Remarkably, two experimental studies found that urban birds raised in a common garden showed attenuated CORT responses to handling stress when compared to their wildland counterparts^27^,^28^, supporting their higher tolerance to human disturbance. On the other hand, the accumulation of apparently contradictory results, even obtained from a single species, might at least partially stem from methodological issues and variability in sources of stress among species^7^. Regarding methodological issues, both baseline and stress-induced CORT concentrations are short-term measures of HPA activity that have often been obtained at different life stages of the studied species^7^. Studies conducted to date have shown no consistent patterns in the within-individual repeatability of these hormonal traits even when measured within a single life history stage and habitat, suggesting that they are rather plastic traits^22^,^35^,^36^. This makes their biological interpretation difficult^37^, including the assessment of differences in allostatic load between urban and rural individuals^7^. In our case, CORT deposited in feathers reflects the duration and amplitude of glucocorticoid secretion during the entire period of feather growth^20^,^21^,^38^ within a single life-history stage, which could span several weeks (see Methods). Both the duration and amplitude of HPA activity are important determinants of CORT_f_, so direct correlations between plasma and feather CORT may not always be expected, especially if the elevation in plasma CORT is relatively modest and brief^21^. However, if urban life actually represents a sustained source of stress for individuals, it is not expected to be a short-live but a long-live stressor which should promote sustained plasma CORT levels for a period long enough to elevate CORT_f_^21^. Estimates of within-individual consistency in CORT_f_ across years are low^22^, indicating it is a plastic hormonal trait that reflects variations in log-term sources of stress and that it could be used to study carry-over effects^22^. In fact, CORT_f_ correlates with subsequent annual survival^23^,^24^ (see below), thus suggesting that it reflects a relatively long-term exposure of individuals to all of the sources of stress that they encounter, and that it could be a more reliable tool for assessing the effects of urbanization on allostatic load than short-term blood CORT measurements.

Regarding variability in sources of stress, Bonier^7^ compiled a variety of biotic and abiotic factors associated with urbanization that could lead to both positive and negative changes in allostatic load. Among them, there are no apparent changes in temperature and food availability that could affect urban owls. Low food availability may cause chronic stress in animals^39^. However, the trophic niche, as assessed through stable isotopes, is nearly identical between urban and rural burrowing owls, and there is a high prey availability in both kinds of habitats (Carrete & Tella, unpublished data). Artificial light should not disturb owls, and in any case may facilitate their foraging at night. Preliminary results suggest that exposure to parasites and diseases neither differ between urban and rural burrowing owls (Carrete & Tella, unpublished data). In addition, the increased breeding densities in urban habitats is not expected to increase allostatic load, since this is a non-territorial species and its mating behavior has been shown to be unaffected by these high densities^11^. Interspecific competition is not expected to increase; the only ecologically similar species preying on the same range of prey (the American falcon *Falco sparverius)* is much scarcer than burrowing owls both in urban and rural areas^6^. Therefore, the only factors that apparently differ are a higher human disturbance and much lower predation risk (see below) in urban than in rural areas. Attending to these sources of stress, an explanation alternative to the selection of individuals related to their fear of humans may explain the identical concentrations of CORT_f_ in urban and rural owls. That is, our results may reflect a replacement of stressful factors, i.e. predators in rural habitats by humans in urban ones. The frequency of exposure to these stressors is however very different: most predation events of adult burrowing owls occur within their burrow nests (authors’ unpub. data), and thus their few encounters with predators would not cause chronic stress (or “sustained physiological stress^40^”) but rather their death; meanwhile, urban birds are constantly facing human presence. Demographic experiments have demonstrated that sustained exposure to predators (or their cues) can have associated physiological stress effects (eg, elevation of glucocorticoid levels) with long-term consequences on birth and survival in free-living animals^40^. Therefore, it seems unreasonable to think that successful city dwellers can live with the permanent activation of their HPA axis^41^ due to daily encounters with humans. Thus, it is more likely that a permanent perturbation factor like human disturbance would be selecting for those individuals that are able to better tolerate humans^6^,^17^.

### Stress and survival

Studies examining relationships between glucocorticoid levels and survival in vertebrates, including birds (e.g.^42^,^43^), showed no less puzzling results than the assessment of variability in allostatic load between rural and urban birds, offering controversial support to the corticostrone-fitness hypothesis^25^,^37^. The variety of responses found between baseline and stress-induced CORT and survival in birds could also be at least partially related to the short-term nature of these hormonal traits and to the variability of the life history stages at which they were measured^25^,^37^. Two recent studies measuring CORT_f_ as a longer term, integrated measure of avian stress physiology have found negative relationships with overwinter survival in two avian species (house sparrows *Passer domesticus*^22^ and northern common eiders *Somateria mollissima borealis*^24^). To our knowledge, ours is the first study assessing differences in CORT-survival relationships between urban and rural individuals, showing contrasting patterns. CORT_f_ showed a quadratic relationship with survival in urban owls, a pattern previously found for baseline CORT in cliff swallows *Petrochelidon pyrrhonota*^42^ that indicates stabilizing rather than directional mortality selection favoring intermediate CORT levels^25^. Although few studies examined non-linear CORT-survival relationships, stabilizing selection makes sense since acute short-term CORT secretion mediates rapid behavioural and physiological modifications that benefit immediate survival, while prolonged stressful situations lead to a long-term allostatic overload that may have detrimental consequences compromising survival^25^,^41^.

Survival probabilities of rural owls were less than half that of urban ones, and contrary to the latter they were unrelated to their CORT_f_ levels. We have strong evidence to suggest that these different patterns result from differences in predation pressure. A lower predation risk is generalized for urban birds^10^, and high predation rates in rural habitats explain differences in breeding densities and the main life-history parameters (breeding success, dispersal) in our study species (Rebolo-Ifrán *et al.*, in prep; authors unpublish data). As breeding burrowing owls have few opportunities to escape from predators entering their deep burrow nests (authors’ unpubl. data), the high rates of predation-caused mortality should be random with respect to their CORT_f_ levels and could mask any relationship between CORT_f_ and survival in rural owls. Our contrasting results comparing urban and rural birds illustrate how different CORT-survival patterns can be obtained within a single species, population, and life-history stage^25^, and how difficult their interpretation can be in the absence of a sufficient knowledge of the ecological constraints modelling the demography of the studied species.

## Conclusions

Our results suggest that urban life does not increase the physiological stress experienced by individuals, supporting the hypothesis that individuals select breeding habitats according to their tolerance to human disturbance as a major challenge faced in urban habitats^6^,^15^. This scenario is in agreement with the adaptive or matching-habitat dispersal hypothesis, which implies a non-random dispersal of individuals resulting in a match between phenotype and environment^18^, a hypothesis thus far scarcely explored^44^. Moreover, the higher survival rates (this study) and breeding success (Rebolo-Ifrán *et al*., in prep) of urban birds have surely contributed to the higher breeding densities currently found in the urban habitat^11^, that together with the high philopatry and the non-random dispersal of individuals between urban and rural habitats generates a genetic structuring of the population (Carrete *et al*. in prep.). Much more research is however needed before we can generalize these results to the many other species that are successfully thriving in urbanized habitats.

## Material and Methods

### Study system and field procedures

We conducted our study in a ca. 5,400 km^2^ area of natural grasslands, pastures, cereal crops and urban areas surrounding the city of Bahía Blanca (Buenos Aires, Argentina), where burrowing owls nest in burrows excavated by the owls themselves or by mammals^11^,^15^,^17^. We surveyed this population between 2006 and 2013, monitoring a total of 1,501 different nests. We defined as urban nests (n = 545) those excavated by owls in private and public gardens, spaces among houses, curbs of streets and along large avenues in the city. Nests are usually within 10–100 m of inhabited buildings. Rural nests (n = 956) were located in the surrounding expanses of natural grasslands and pastures devoted to wide-ranging livestock and low-intensive cereal crops, where human presence and activities are minimal^6^. There is no clear habitat interface between urban and rural habitats, since urbanized areas are immediately surrounded by rural ones.

For this study, we captured 183 different breeding adults in 2006, 2008 and 2009 (74 in rural and 109 in urban nests) during the chick-rearing period (late November to early January). Birds were marked with plastic rings with a unique alphanumeric code that could be read at a distance using a telescope. Blood samples (0.1 ml) were collected for molecular sexing (see^11^), while one external tail feather was plucked for CORT_f_ determination (see below). Resightings of marked birds were obtained during intensive population monitoring from 2007 to 2012, surveying all known breeding sites as well as areas of suitable habitat not previously occupied to detect new breeding sites.

We were able to experimentally measure flight initiation distance (FID, i.e. the distance at which individuals flee when approached by a human), of 121 out of the 183 individuals sampled for CORT_f_, following the standard protocol used in previous studies^15^,^17^. Briefly, all FIDs were also measured during the chick-rearing period, by walking towards focal individuals following a direct trajectory, with no obstacles blocking the bird and the observer and at a constant speed of 0.5 m/s. FIDs were measured in fine weather, avoiding the hottest hours of the day, and rainy or windy days. All tested owls were undisturbed and resting close to or at the entrance of their nests during daytime. The distance between the observer and the flushing bird was measured using a laser telemeter (range: 10–1300 m) or counting paces for distances of less than 10 m. We used for analyses both single and average values of FID (when more than one measure was obtained from a single individual in the same or different years), given that this behavior is highly repeatable within breeding seasons (r = 0.84–0.92) and across the lifespan (r = 0.90–0.96) of burrowing owls^15^,^17^.

### Feather corticosterone

We estimated the stress experienced by birds by measuring CORT concentration in feathers following Bortolotti *et al*.^20^. Contrary to blood measures, CORT_f_ reflects the HPA activity of an individual within a time frame of days or weeks (depending on the moult speed of the studied species). Thus, this hormonal trait integrates not only the intensity of the physiological response but also how long CORT is elevated within the bloodstream, and the frequency of exposure to stressors^20^,^21^,^38^. In the burrowing owl, no information is available about the growth rate of feathers and growth bars are not conspicuous as in other species (authors’ unpublished data). Estimates for other owls show that flight feathers can growth 1.9–3.1 mm/day^45^, so a typical 95 mm tail feather from a burrowing owl may be fully grown after ca. 30–50 days. Although this may not be an accurate estimation, CORT_f_ can reflect the stress experienced by individuals during a time frame of at least 3–4 weeks corresponding to the pre-breeding moult period of adult birds. Burrowing owls are sedentary and breeders stay in their territories along the year in the study area (author’s unpubl. data). Therefore, the studied individuals were potentially exposed to the same stressors associated to their territories across the annual cycle.

To quantify CORT_f_, we first measured the length of each feather after removing the calamus^46^. Then we cut the feather vanes into small pieces (<5 mm) with scissors and placed in a glass vial with 10 mL of methanol (HPLC grade, VWR International, Mississauga, ON) for CORT_f_ extraction^20^,^38^ .Vials were placed in a sonicating water bath at room temperature for 30 min, followed by incubation at 50 °C overnight in a shaking water bath. Using vacuum filtration, the methanol containing the hormones was separated from the feathers. Vials with the methanol extract were placed open in a 50 °C water bath in a fume hood under air until they were completely dry. When the evaporation of the samples was complete, the extract residues were reconstituted in 600 mL of phosphate buffer system (0.05 M, pH 7.6) and frozen at −20 °C until CORT measurement by radioimmunoassay. We assessed the extraction efficiency by including feather samples spiked with a small amount (*c.* 4000 dpm) of 3H-corticosterone. The coefficient of variation for five different batches ranges from 5.31 (within essay) to 8.32 (between essays). Greater than 92% of the radioactivity was recoverable in the reconstituted samples. CORT_f_ values are expressed as a function of feather length (pg mm-1) following Bortolotti *et al.*^20^,^38^.

### Statistical analysis

We used generalized linear models (GLM, using the identity link function and normal error distribution) to assess if FID (log-transformed) differed between our sampled rural and urban breeding owls, while controlling for sex, a result that was previously found using different datasets of individuals^6^,^15^,^17^. We were able to sample both members of the breeding pair in only 25% of the territories, so we could not control for a potential territory effect fitted as a random term. However, a previous study conducted in the same owl population, using larger sample sizes, showed just a slight effect of territory and results were nearly identical when it was not fitted as a random term^15^. The year(s) when FID was measured for each individual was not controlled for given the high consistency in this behavioural trait across the lifespan of individuals (see above). The same GLM structure was then used for assessing differences in CORT_f_ (log-transformed) between urban and rural birds and its relationship with FID, while controlling for potential sources of variability (sex and sampling year). In this case, we controlled for sampling year since CORT_f_ of individuals may vary across years due to yearly variations in the kind and intensity of sources of stress^22^. All models were fitted using R 3.0.3.

### Survival analysis

We analyzed encounter histories of the 183 individuals sampled in 2006, 2008 and 2009 and resighted (or not) from 2007 to 2012 to test for habitat and CORT_f_ effects on survival. This monitoring period is longer than the average lifespan of adult burrowing owls^15^, and no individuals changed breeding habitat (urban or rural) during the study. Survival (ϕ) and resighting probabilities (*p*) were estimated simultaneously by maximum-likelihood procedures^47^ and models were built using the program MARK^48^. We conducted capture-recapture analyses in two steps: first we tested for temporal and habitat effects on both resighting and survival probabilities; then, we selected the best structure of the parameters to specifically test for CORT effects on subsequent survival probabilities (i.e, only first year survival after sampling was modeled as a function of the CORT_f_ covariate, including linear and quadratic effects). Exploratory analyses did not detect a sex effect on survival or resighting probabilities, nor was there a strong sex effect on CORT_f_ (see results), so we did not include sex in final models to avoid overparameterization. The goodness-of-fit of the general Cormack-Jolly-Seber model by group (i.e, urban and rural birds; ϕ_t*g_ *p*_t*g_) was assessed using program U-CARE 2.3.2^49^. Model selection was based on Akaike’s Information Criterion adjusted for the effective sample size (AICc^50^). Models differing less than 2 AICc points were considered equivalent^50^. We also calculated for each model the Akaike weight (w), as an index of its relative plausibility^50^.

### Ethic statements

Capture, banding and FID measures of Burrowing owls were conducted under permits and following the protocols approved by the Argentinean wildlife agency (22500-4102/09), the Ethic Committee of CSIC (CEBA-EBD-11-28), and the owners of private properties, in accordance with the approved guidelines.

## Additional Information

**How to cite this article**: Rebolo-Ifrán, N. *et al.* Links between fear of humans, stress and survival support a non-random distribution of birds among urban and rural habitats. *Sci. Rep.* **5**, 13723; doi: 10.1038/srep13723 (2015).

## Figures and Tables

**Table 1:** Models obtained to compare FID and feather CORT (CORT_f_) levels between urban and rural birds (n = 121 individuals).

Dependent variable	Independent variables	F	df	P	Dependent variable	Independent variables	F	df	P
(log)FID	Habitat	115.20	1, 118	0.0001	(log)CORT_f_	Habitat	0.34	1, 116	0.5591
	Sex	1.45	1, 118	0.2311		Sex	3.62	1, 116	0.0594
						Year	0.28	1, 116	0.5953
						FID	0.38	1, 116	0.5398
Adjusted-R^2^: 0.49					Adjusted-R^2^: 0.003				

**Table 2:** Modeling survival (ϕ) and recapture probabilities (*p*) of 183 adult burrowing owls. AICc: Akaike’s information criterion adjusted for effective sample size, ΔAICc: AICc difference between the current model and that with the lowest AICc value; w_i_: Akaike’s weight, NP: number of estimable parameters, deviance: relative deviance. Model notation: “+”: parallel variation, additive effect; “x”: interaction; “.” = constant (i.e. no effects considered); “habitat” = different parameters for urban and rural birds; “time” = between year differences.

Model	Model structure	AICc	ΔAICc	w_i_	NP	deviance
1	ϕ (habitat) *p* (time)	409.229	0.00	0.528	8	392.702
2	ϕ (habitat × time) *p* (time)	411.303	2.07	0.187	17	374.985
3	ϕ (habitat + time) *p* (time)	412.345	3.12	0.111	13	384.987
4	ϕ (habitat × time) *p* (habitat + time)	412.794	3.56	0.089	18	374.193
5	ϕ (habitat × time) *p* (habitat)	414.613	5.38	0.036	14	385.040
6	ϕ (habitat × time) *p* (.)	414.754	5.52	0.033	13	387.396
7	ϕ (habitat × time) *p* (time)	416.156	6.93	0.017	20	372.938
8	ϕ (.) *p* (time)	433.641	24.41	0.000	7	419.232
9	ϕ (t) *p* (time)	434.354	25.12	0.000	11	411.376

**Table 3:** Modeling feather CORT (CORT_f_) effects on subsequent annual survival (ϕ) of urban (n = 109) and rural (n = 74) adult burrowing owls. In all models, resighting probabilities (*p*) varied over time and survival after first year of CORT_f_ sampling was considered to be constant over time and different for rural and urban birds. Model notation: “-”: constant (i.e. no CORT_f_ effect); CORT_f_: linear CORT_f_ effect; CORT_f_ + CORT_f_^2^: quadratic CORT_f_ effect. All other notations as in Table 1.

Model	ϕ _urban_	ϕ _rural_	AICc	ΔAICc	w_i_	NP	deviance
1a	CORT_f _+ CORT_f_^2^	CORT_f_	403.89	0.00	0.39	13	376.54
1b	CORT_f _+ CORT_f_^2^	—	405.79	1.90	0.15	12	380.63
1c	CORT_f _+ CORT_f_^2^	CORT_f _+ CORT_f_^2^	406.07	2.18	0.13	14	376.50
1d	CORT_f_	CORT_f_	406.31	2.41	0.12	12	381.15
1e	—	CORT_f_	406.95	3.06	0.08	11	383.98
1f	CORT_f_	—	408.33	4.43	0.04	11	385.35
1g	CORT_f_	CORT_f _+ CORT_f_^2^	408.48	4.58	0.04	13	381.12
1h	—	fCORT_ _+ fCORT ^2^	409.12	5.23	0.03	12	383.96
1i	—	—	409.28	5.38	0.03	10	388.46
